# A microbial expression system for high-level production of scFv HIV-neutralizing antibody fragments in *Escherichia coli*

**DOI:** 10.1007/s00253-019-10145-1

**Published:** 2019-10-22

**Authors:** Marloes L. C. Petrus, Lukas A. Kiefer, Pranav Puri, Evert Heemskerk, Michael S. Seaman, Dan H. Barouch, Sagrario Arias, Gilles P. van Wezel, Menzo Havenga

**Affiliations:** 1 Molecular Biotechnology, Institute of Biology, Leiden University, Sylviusweg 72, 2333 BE Leiden, The Netherlands; 2 Batavia Biosciences B.V., Zernikedreef 16, 2333 CL Leiden, The Netherlands; 3 Center for Virology and Vaccine Research, Beth Israel Deaconess Medical Center, 3 Blackfan Circle, Boston, MA 02115 USA

**Keywords:** Protein production, Rhamnose-inducible promoter, Antibody fragments, Antibody purification, HIV

## Abstract

Monoclonal antibodies (mABs) are of great biopharmaceutical importance for the diagnosis and treatment of diseases. However, their production in mammalian expression hosts usually requires extensive production times and is expensive. *Escherichia coli* has become a new platform for production of functional small antibody fragment variants. In this study, we have used a rhamnose-inducible expression system that allows precise control of protein expression levels. The system was first evaluated for the cytoplasmic production of super folder green fluorescence protein (sfGFP) in various production platforms and then for the periplasmic production of the anti-HIV single-chain variable antibody fragment (scFv) of PGT135. Anti-HIV broadly neutralizing antibodies, like PGT135, have potential for clinical use to prevent HIV transmission, to promote immune responses and to eradicate infected cells. Different concentrations of L-rhamnose resulted in the controlled production of both sfGFP and scFv PGT135 antibody. In addition, by optimizing the culture conditions, the amount of scFv PGT135 antibody that was expressed soluble or as inclusions bodies could be modulated. The proteins were produced in batch bioreactors, with yields of 4.9 g/L for sfGFP and 0.8 g/L for scFv. The functionality of the purified antibodies was demonstrated by their ability to neutralize a panel of different HIV variants in vitro. We expect that this expression system will prove very useful for the development of a more cost-effective production process for proteins and antibody fragments in microbial cells.

**Electronic supplementary material:**

The online version of this article (10.1007/s00253-019-10145-1) contains supplementary material, which is available to authorized users.

## Introduction

Over the past years, the development of the antibody therapeutic field has made significant progress, driving a sustained increase in the number of antibodies that are granted their first marketing approvals each year, with a new record of 10 monoclonal antibody (mAb) therapeutics approved in 2017 (Kaplon and Reichert 2018). The potential impact of monoclonal antibodies on the entire pharmaceutical industry is also illustrated by their global sales in 2017, which grossed $108 billion, and it is expected to continue growing during the upcoming years (Grilo and Mantalaris 2019). The remarkable success of mAbs comes from their high level of target selectivity and their applicability to a wide range of diseases as for instance, in oncology and neurodegenerative or autoimmune disorders and their widespread use in diagnosis applications in the fields of radioimmunotherapy and radiology (Scott et al. 2012; Smilek et al. 2014).

Currently, most of the mAbs are produced using mammalian cells, which can perform human-like N-glycosylation as posttranslational modifications. However, a disadvantage of using mammalian cell culture for heterologous protein production is an inconvenient and time-consuming production process, which is sometimes difficult to scale up. Other issues relate to typically low product yields and growth rates, the risk of viral contamination, and the need for complex growth media (Spadiut et al. 2014; Tripathi and Shrivastava 2018; He et al. 2019). These disadvantages in the production process lead to high production costs and limit the wide use of mAbs as drugs (Chames et al. 2009; Spadiut et al. 2014). Nowadays, some of these disadvantages are tackled by genetically engineering mammalian stable cell lines that can express higher titers of the target protein within shorter timelines but still, alternative production platforms that can generate high-quality products in shorter times are always sought.

In view of these issues, there is an increasing demand for microbial platforms as alternative production platform for fast and cost-effective production of proteins and mAbs. *Escherichia coli* is one of the preferred organisms since it can be easily genetically manipulated and high cell densities can be reached using inexpensive media, free of animal components, in a short time span (Frenzel et al. 2013; Spadiut et al. 2014; Jozala et al. 2016). However, *E. coli* lacks the eukaryotic posttranslational modification system needed for the N-glycosylation of the Fc domain of full-size antibodies, and thus, the correct folding of these large antibody molecules can be problematic, possibly also affecting serum half-life. However, it has been recently shown that various antibody fragments retain the binding activity of the full-length antibody without the presence of the Fc domain (Nelson 2010; Baeshen et al. 2015). Owing to the lack of glycosylation and their smaller size, these antibody fragments can be readily produced in active form via prokaryotic expression systems (Nelson 2010; Spadiut et al. 2014).

The single-chain variable fragments (scFv) represent a class of antibody fragments suitable for expression in *E. coli*. These scFv fragments consist of a single polypeptide in which the variable regions of light and heavy immunoglobulin chains are joined by a flexible peptide linker (Nelson 2010; Ahmad et al. 2012). ScFv antibody fragments are very attractive in drug development due to their small size in comparison with full-length antibodies and their drug-delivery characteristics; this makes them more suitable for certain clinical applications. For instance, they enable enhanced tissue penetration, which is highly useful in oncology or for treatment of neurodegenerative diseases; as well as faster blood clearance and lower retention times in non-target tissue, which can be beneficial, for example, in radio immunotherapy and radiology applications (Nelson 2010; Ahmad et al. 2012). Interestingly, the most clinically advanced humanized scFv antibody fragment Brolucizumab (Novartis) against wet age-related macular degeneration (AMD) has shown promising results from a 2-year phase III HAWK and HARRIER study and it is expressed in *E. coli* (Dugel et al. 2017; Althoff and Wolf 2018; Kaplon and Reichert 2018).

Besides the lack of glycosylation, the main difficulty when expressing antibody fragments in prokaryotic cells is the correct folding of the protein and the formation of essential disulfide bridges. Directing the antibodies to the oxidizing and chaperone-rich environment of the periplasm of *E. coli* has been the most successful strategy in the production of correctly folded recombinant proteins. Additional advantages of periplasmic secretion are reduced costs for downstream processing due to lower levels of total protein and less proteolytic degradation in the periplasmic fraction (Ahmad et al. 2012; Jalalirad 2013; Baeshen et al. 2015). However, the protein secretion machinery is easily saturated resulting in cell toxicity and reduced production yields. Thus, gene expression levels should be tightly balanced with the secretion capacity to optimize antibody production or optimized engineered strains with better secretion capacity should be used (Schlegel et al. 2013; Gaciarz et al. 2016).

In this study, we use the tightly regulated L-rhamnose promoter from the P*rhaBAD* operon (Giacalone et al. 2006; Kelly et al. 2016) for the precise control of protein expression. The rhamnose regulon consist of a rhamnose transporter gene *rhaT*, the genes for rhamnose catabolism *rhaB*, *rhaA*, and *rhaD* and the regulation genes *rhaR* and *rhaS*. The regulon reacts to the presence of L-rhamnose by activating the transcriptional regulator RhaR to induce expression of *rhaR* and *rhaS*. In turn, accumulation of RhaS results in the L-rhamnose dependent induction of both the p*rhaBAD* and the p*rhaT* promoters, thus activating rhamnose catabolism (Giacalone et al. 2006; Marschall et al. 2017).

The super folder green fluorescent protein (sfGFP) was first used to study the inducibility of the promoter and to determine if the population of cells expressing sfGFP was homogeneous. Additionally, an scFv antibody fragment based on the full IgG HIV broadly neutralizing antibody PGT135 was designed and used as an example for expression of a clinically relevant and functional antibody fragment in *E. coli*. PGT135 antibody was originally identified from the antibody repertoire of a HIV-1 infected donor with remarkably broad and potent neutralizing responses and is able to neutralize about 35% of the clade C viruses tested (Walker et al. 2011). Considering that clade C is responsible for more than 50% of all HIV infections worldwide, PGT135, together with other neutralizing antibodies, might become promising biopharmaceutical candidates to prevent HIV transmission, promote immune responses, and eradicate infected cells (Walker et al. 2011).

In this study, both proteins were successfully expressed using the pSAR-2 expression vector. The sfGFP protein was produced in the cytoplasm while the scFv was translocated into the periplasm of *E. coli* to allow proper folding. In batch bioreactors without any supplementation of extra feed, 4.9 g/L of sfGFP and 0.8 g/L of scFv were produced in total cells. A total amount of 54 mg tag-free PGT135 scFv antibody fragment could be purified per liter of culture. *In vitro* HIV neutralization assays were utilized to demonstrate the functionality of the PGT135 scFv antibody fragment. Together these data show that the pSAR-2 rhamnose-inducible vector can be an important tool for the development of a more cost-effective protein production system in microbial cells.

## Materials and methods

### Bacterial strains, plasmids, and growth conditions

*Escherichia coli* JM109 (Sambrook et al. 1989) was used for routine cloning procedures and *E. coli* BL21 (Novagen/Merck, Darmstadt, Germany ) for expression of the recombinant proteins. For cloning procedures, cells were grown in low salt Luria-Bertani broth (LB) or on low salt LB agar plates at 37 °C and selection pressure was applied by the addition of 25 μM of Zeocin. The plasmid vector pSAR-2 was completely synthetically manufactured (Invitrogen GeneArt Gene Synthesis, Regensburg, Germany). Plasmid pSAR-2 was designed on the basis of the plasmid pD881-SR (DNA2.0, Menlo Park, California) and modified extensively to contain (i) a multiple cloning site, (ii) an alternative origin of replication (pBR322), and (iii) a zeocin selection marker instead of the kanamycin selection marker. The *Nde*I*/Bam*HI restriction sites present in the multiple cloning site were used for inserting the genes of interest. The gene coding for super-folded green fluorescent protein (sfGFP) (Pédelacq et al. 2006) and for PGT135 scFv antibody fragment was codon optimized and synthesized by Invitrogen GeneArt Gene Synthesis (Regensburg, Germany) and cloned into the vector. The sequence of the PGT135 scFv fragment, including a PelB leader sequence for protein translocation to the periplasm, was designed and kindly provided by Ragon Institute (Cambridge, MA, USA). This antibody fragment was designed based on the PGT135 antibody sequence (AEN14416.1) in which the light (VL) and heavy (VH) chain variable regions were linked by a 20 amino acids string consisting of glycine and serine residues (GGGGSGGGGSGGGGSGGGGS) (Walker et al. 2011). Before transformation into *E. coli* BL21, plasmid minipreps were performed using the GeneJet Plasmid Miniprep Kit (ThermoScientific, Waltham, MA, USA). All constructs were confirmed by restriction analysis and sequencing.

The Genbank accession numbers for the codon-optimized genes are MN146014 for sfGFP and MN146015 for scFV_PGT135.

### sfGFP expression

For expression of sfGFP in shaker flasks, *E. coli* strains were inoculated from glycerol stocks into 5 ml of low salt LB with 25 μM Zeocin and 0.5% glucose and grown overnight at 37 °C and 250 rpm. The next morning cultures were diluted 100× in 500 mL shaker flasks containing 100 ml of TB expression medium (1.2% animal free soytone (Difco), 2.4% yeast extract (Oxoid), 0.4% glycerol, 17 mM KH_2_PO_4_, and 72 mM K_2_HPO_4_) with 50 μM Zeocin. When the OD_600_ reached 0.6–0.8, cultures were induced with L-rhamnose as indicated, and growth was continued at 37 °C and 250 rpm. For optimization of expression conditions in TubeSpin® Bioreactors (50 ml), 5–10 ml of TB expression media were used. Preculture and culture conditions were prepared as indicated for shaker flasks. For sfGFP expression in 250 ml UltraYield™ flasks (Thomson Instrument Company, San Diego, Ca, USA), (pre-)cultures were prepared and grown as described for the regular shaker flasks, except that cultures had a volume of 50 mL medium and were induced at an OD_600nm_ of 0.5 with L-rhamnose to a final concentration of 10 mM.

A bioreactor with 5 L TB medium and 1 mL/L Antifoam P2000 without Zeocin was used for large scale expression of sfGFP. The bioreactor was controlled at 37 °C and induction was performed at an OD_600nm_ of 0.6–0.8 by addition of L-rhamnose to a final concentration of 10 mM. Aeration of the cultures was based on a tripartite cascade in order to maintain a dissolved oxygen concentration (DO) of 30%. For that purpose, stirring was increased from + 276 rpm up to 750 rpm; a constant overlay flow (headspace aeration) of a gas mixture of air and pure oxygen (20–100% O_2_, 4 L/min) was applied; and sparged aeration with pure oxygen (O_2_, 0–10 L/min) was used. pH was controlled at 7.2 ± 0.02 by addition of acid (HCl) and base (NaOH).

### Analysis of sfGFP expression

To quantify sfGFP expression, samples were normalized to 1 mL culture with an OD_600nm_ of 1.0, centrifuged and resuspended in 1 mL of BugBuster® Protein Extraction Reagent with 5 μL protease-inhibitor cocktail set III and 1 μL of Lysonase™ Bioprocessing Agent (all from Merck, Darmstadt, Germany). Cells were lysed for 20 min at room temperature (RT) and insoluble debris was pelleted by centrifugation. The lysates were serially diluted using phosphate buffer saline (PBS) and a standard curve of commercially available his-tagged GFP (Life Technologies by ThermoScientific, Waltham, MA, USA) was prepared. A BMG Labtech FLUOstar® OPTIMA Microplate Reader (BMG Labtech, Offenburg, Germany) was used to measure fluorescence in a black 96-well microplate. The device was set to 485 nm ± 12 nm excitation and 520 nm emission wavelengths with 15 flashes per well and double orbital mixing at 300 rpm for 25 s.

Cultures in shaker flasks for flow cytometric analysis (FACs) were performed as mentioned above, although 50 mL shaker flasks with 10 mL of medium were used. From this cell suspension, 100 μL was used for FACS analysis on a BD LSR Fortessa X-20 instrument (BD BioSciences, San Jose, CA, USA) equipped with a 488-nm laser. For each culture, at least 100,000 events were recorded in the specified gate using the 530/30 filter in FITC channel. Flowing Software was used for data analysis and processing.

### scFv PGT135 antibody fragment expression

For expression of the scFv PGT135 antibody fragment in shaker flasks, (pre-)cultures were prepared as for the expression of sfGFP. For initial screening of expression conditions, L-rhamnose concentrations ranged from 0.1–15 mM, while for quantification and production experiments concentrations of 3 mM, 10 mM, and 15 mM were selected. To allow time for protein expression and secretion, growth was continued for 21 h at 30 °C or for 48 h at 25 °C as indicated. Normally, 50 mL aliquots of cell culture were harvested by centrifugation (5.000*g*, 10 min, 4 °C) and pellets were frozen at − 20 °C.

Before fermenter cultures were performed, the time of induction (OD_600nm_) and harvest time were optimized by using Ultrayield shaker flasks as mentioned for the sfGFP protein. Next, a large scale expression experiment of PGT135 scFv antibody fragment was performed, and precultures were prepared and inoculated 1:100 as above. Two different bioreactor setups at 0.7 L scale and in duplicates were used, and cultured either at 30 °C or 25 °C. All fermentations were based on a working volume of 500 mL TB medium containing 50 μg/mL Zeocin™ and 1 mL/L Antifoam P2000. Incubation prior to induction was carried out at 37 °C and two Rushton impellers were used for agitation at 800 rpm. Induction was performed at an OD_600nm_ of 2–3 and stirring was increased to 1000 rpm. For the first bioreactor setup, the incubation temperature was decreased to 30 °C after induction with 15 mM of L-rhamnose, while for the second setup, temperature was lowered to 25 °C after induction with 10 mM of L-rhamnose. During the whole fermentation, a microsparger delivered a constant air flow (0.5 L/min) of a gas mixture regulating between air and oxygen, thereby maintaining a dissolved oxygen concentration of at least 30% in combination with the 800–1000 rpm agitation mentioned above. The pH of the media was controlled at 7.0 ± 0.1 by addition of acid (HCl) and/or base (NaOH). The bioreactors were harvested after 7 h for the first setup at 30 °C, and after 18 h for the second setup at 25 °C.

### Sample preparation and western blot analysis

For analysis of both sfGFP production and PGT135 scFv expression, cell pellets were lysed as described for the analysis of sfGFP fluorescence. The total cell protein fraction was directly analyzed or separated into a soluble and insoluble protein fraction by centrifugation at 16.000×*g* for 20 min at 4 °C. The pelleted insoluble fraction was resuspended in the original volume with B-PER^TM^ Bacterial protein extraction reagent (Thermo Scientific, Waltham, MA, USA) followed by incubation for 20 min at RT.

A Quick Start™ Bradford Protein Assay (Bio-Rad, Hercules, CA, USA) was performed before loading the samples on a NuPAGE® Novex® 12% Bis-Tris Gel (Life Technologies by Thermo Scientific, Waltham, MA, USA). After SDS-PAGE, proteins were electro transferred to PVDF iBlot™ 2 Transfer Stacks (Life Technologies by Thermo Scientific, Waltham, MA, USA) using an iBlot^TM^ 2 device (Invitrogen, Regensburg, Germany) run at 20 V for 1 min, 23 V for 4 min, and 25 V for 2 min. The membrane was blocked overnight with 5% non-fat dry milk blotting grade blocker (Bio-Rad, Hercules, CA, USA) in PBS and washed three times with 0.1% Tween20 in PBS. The membrane was then incubated in 1 μg/mL Protein L –horseradish peroxidase (HRP) conjugate (GenScript, Piscataway, NJ, USA) in PBS and the washing steps were repeated. Blots were developed with TMB Enhanced One-Component HRP substrate (Sigma-Aldrich, Vienna, Austria) and washed with MilliQ water. Data were analyzed using Image Lab software (Version 4.0.1, Bio-Rad, Hercules, CA, USA). Different amounts of commercial anti-HIV gp120 scFv PGT135-His-tagged antibody (0.5 mg/mL, > 70% purity, Creative Biolabs, Shirley, NY, USA) served as internal standards. Two biological replicates of each sample were used.

### Purification of scFv PGT135 antibody fragment

For semi large scale purifications, PGT135 scFv antibody fragment was isolated from the soluble protein fraction originating from the lysate (lysis as described for sfGFP analysis) of cell pellets of 50 mL culture. This fraction was filtered through a 0.45-μm syringe filter (Pall, Port Washington, NY, USA) and diluted with an equal amount of binding buffer (100 mM sodium phosphate, 150 mM sodium chloride, pH 7.2). Affinity chromatography was performed using an ÄKTA pure chromatography system (GE Healthcare Life Sciences, Chicago, IL, USA). A pre-packed 5 mL Capto L column (GE Healthcare Life Sciences, Chicago, IL, USA) was equilibrated with binding buffer and 15.5 mL of diluted soluble protein was applied at a flow rate of 2 mL/min. After washing the column with binding buffer, PGT135 scFv was recovered using elution buffer (50 mM Glycine, 50 mM Citrate, pH 2.0) and immediately pH neutralized with neutralization buffer (1 M Tris, pH 8). Fractions containing the PGT135 scFv antibody fragment were desalted using a 2-ml Slide-A-Lyzer™ MINI Dialysis Device with a 10-K molecular weight cut-off (Thermo Scientific, Waltham, MA, USA) by a two-step overnight buffer exchange with PBS. Where necessary, purified fractions were concentrated with Amicon Ultra-15 Centrifugal Filter Units with a 10-K molecular weight cut-off (Merck, Darmstadt, Germany).

### HIV neutralization assay

Neutralization activity of (partially purified) PGT135 scFv samples and a pure full-length IgG control were determined using the luciferase-based HIV-1 neutralization assay in TZM.bl cells as previously described (Montefiori 2009; Sarzotti-Kelsoe et al. 2014). This assay measures the reduction in luciferase reporter gene expression in TZM-bl cells following a single round of virus infection. Antibodies were tested using a primary concentration of 25 μg/ml with a 5-fold dilution series in duplicate wells (96-well flat bottom plate) in 10% DMEM growth media (100 μl/well). Virus was added to each well in a volume of 50 μl, and the plates were incubated for 1 h at 37 °C. TZM.bl cells were then added (1 × 10^4^/well in 100 μl volume) in 10% DMEM growth medium containing DEAE-Dextran (11 μg/ml). Following a 48-h incubation period, luminescence was measured using Bright-Glo luciferase reagent (Promega, Madison, WI). Samples were tested against a multiclade panel of 19 HIV-1 Env pseudoviruses with known sensitivity to PGT135 antibody. Murine leukemia virus (MuLV) was used as a negative control virus for all assays. Concentrations of antibody that inhibited 50% or 80% of virus infectivity (IC50 and IC80, respectively) were also determined based on molarity, to normalize for differences in molecular weight between the scFv antibody fragments and the full-length IgG antibody. All assays were performed in a laboratory meeting GCLP standards.

## Results

### Characterization of pSAR-2 expression vector using sfGFP

**Fig. 1:**
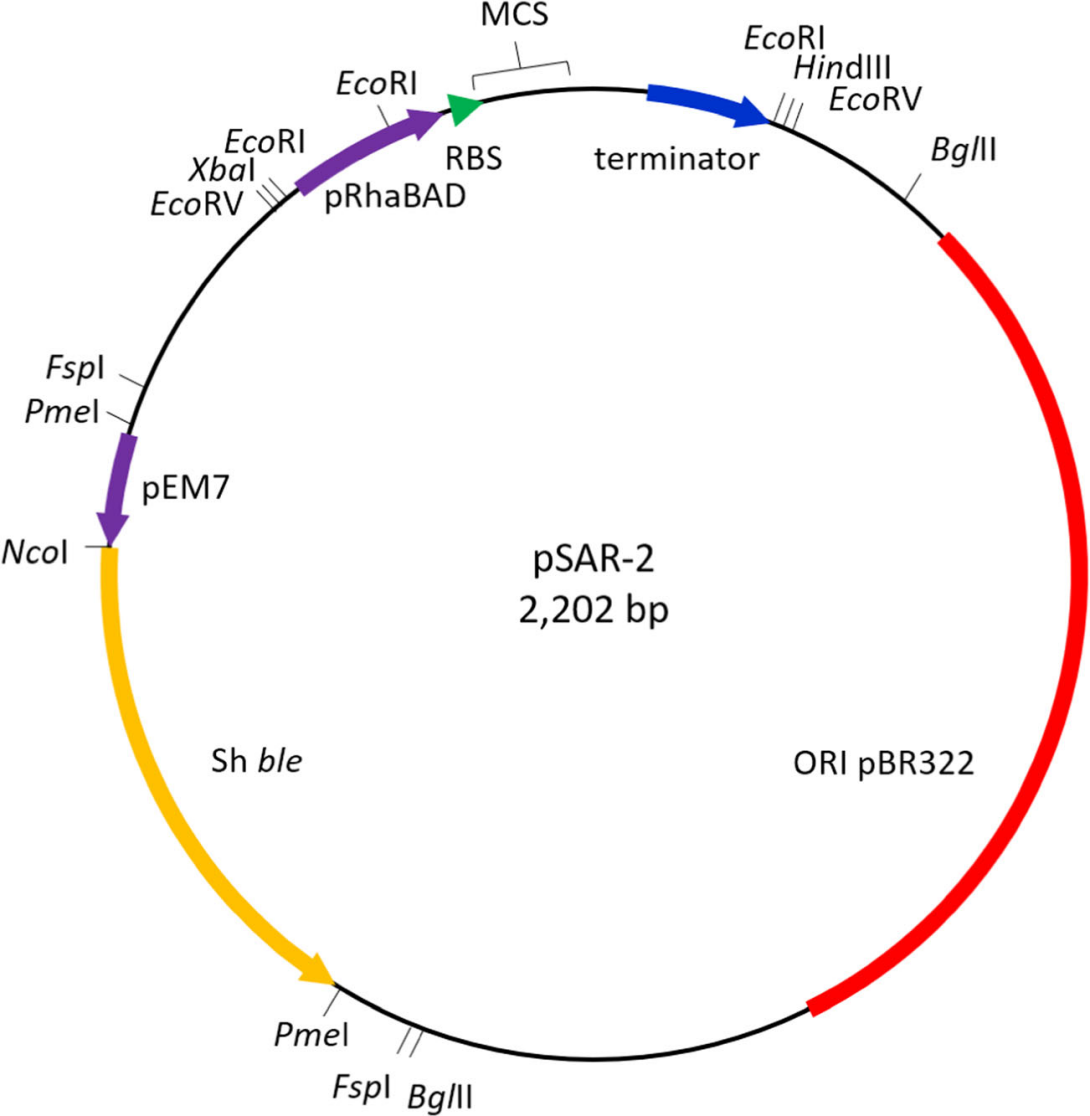
Plasmid map of expression vector pSAR-2 showing the most relevant vector elements. Important restriction enzyme sites and respective transcription sense of the pSAR-2 components are indicated. The multicloning site (MCS) has been modified from that of pUC18 to facilitate the cloning of the gene of interest. Each of the plasmid elements is flanked by restriction sites to allow exchange to the different elements if required. pRhaBAD, promoter of the rhamnose operon; RBS, ribosome binding site; MCS, multiple cloning site; terminator, transcription terminator region; ORI, origin of replication; Sh *ble*, Bleomycin family of antibiotics resistance gene; pEM7, EM-7 promoter

Under this study, we utilized a rhamnose-inducible expression vector (pSAR-2) to produce proteins that require tight control of transcription levels at high yields. The pSAR-2 plasmid is a medium copy vector that contains the pBR322 *ori* without the *rop* gene (Balbás et al. 1986; Cesareni et al. 1991; Giacalone et al. 2006). The plasmid consists of flexible building blocks in which the different elements can be easily replaced due to flanking restriction enzyme sites (Fig. 1). The Sh *ble* gene under the EM7 promoter (Lane et al. 2007) on the vector confers resistance to Zeocin, a broad-spectrum antibiotic of the bleomycin family accepted for use in manufacturing of clinical products (Drocourt et al. 1990; Mignon et al. 2015). To insert genes in the expression vector, they need to be cloned by using the *Nde*I restriction site, overlapping with the ATG start codon, allowing fusion directly behind the RBS at an optimal distance (Chen et al. 1994; Berwal et al. 2010). Immediately downstream a polylinker derived and modified from the pUC18 vector (Norrander et al. 1983) is present for cloning purposes. This vector can be used in any *E. coli* background strain as it does not rely on the expression of strain-specific RNA polymerases.

The super folder green fluorescent protein (sfGFP) was initially used as a model protein for the induction experiments and to probe the tunability of the rhamnose promoter for protein expression (Pédelacq et al. 2006). We cloned the synthetically produced and codon-optimized sfGFP gene (Genbank Accession MN146014) into the pSAR-2 vector and transformed the construct to *E. coli* BL21 cells for expression. The recombinant cells were grown in shaker flasks containing 100 ml TB medium with Zeocin and induced with a range of rhamnose concentrations when the OD_600nm_ reached 0.6–0.8. Samples were taken 5 and 7 h after induction and expression of sfGFP was monitored using whole cell lysate fluorescence measurements. Commercially available sfGFP-His_6_ protein was used for quantification of sfGFP based on fluorescence measurements.

**Fig. 2:**
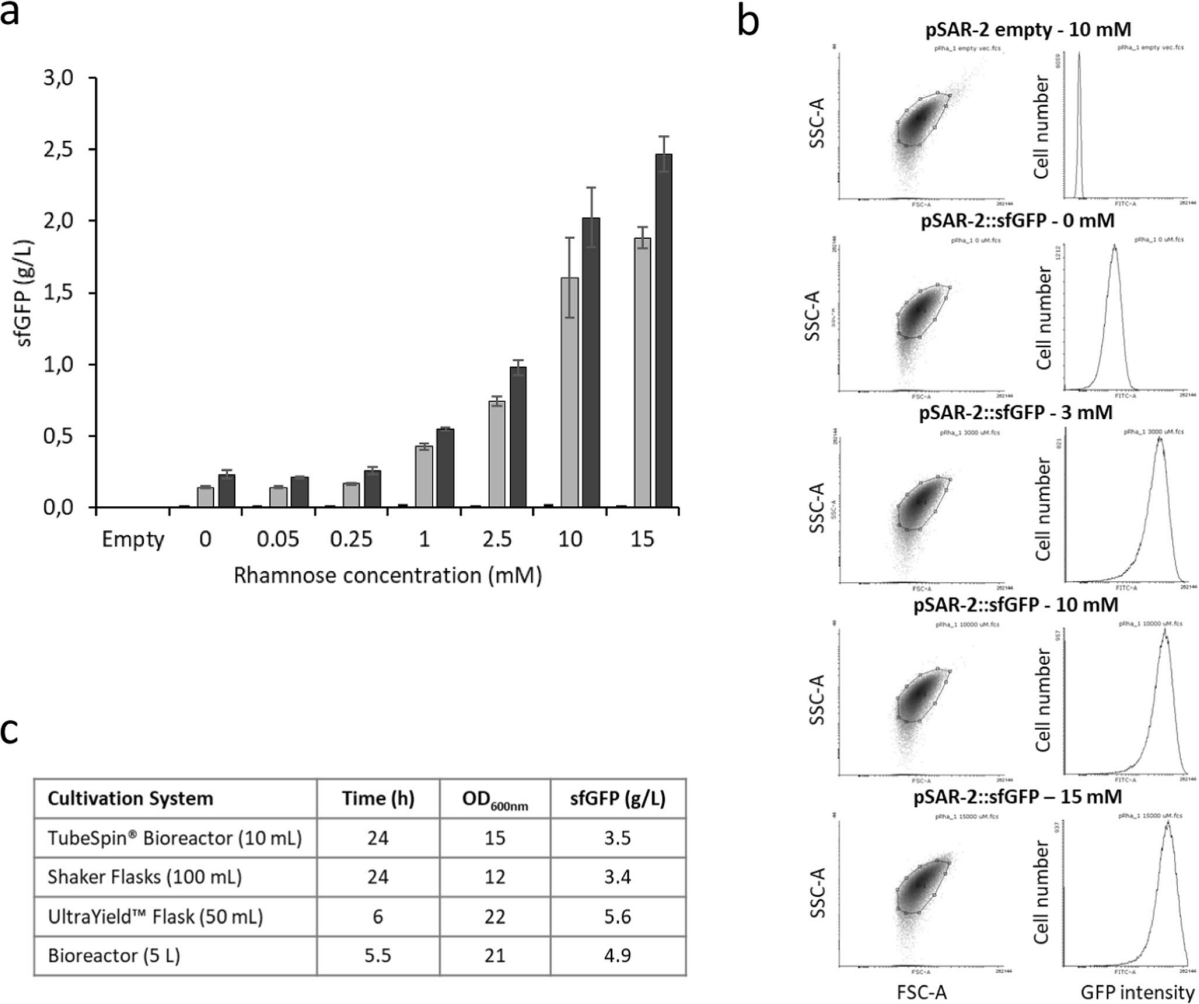
Controlled induction of sfGFP expression. **a** Fluorescence quantification of sfGFP production with increasing amounts of rhamnose in shaker flasks at different time points: pre-induction (white), 5 h (gray), or 7 h (black) after induction. The *E. coli* pSAR-2 strain without sfGFP insert is indicated as “Empty”. Error bars indicate standard deviations of two biological replicates. **b** Flow cytometry analysis (FACS) data of *E. coli* BL21 cells with pSAR-2 empty and pSAR-2::sfGFP vector expressing sfGFP, 4 h after induction with different amounts of rhamnose in shaker flasks. Left plots showing cell granularity by forward scatter (FSC-A, *x*-axis) and side scatter (SSC-A, *y*-axis) per cell. Right histograms showing the number of cells per mean GFP fluorescence intensity (au). **c** Scalability of sfGFP expression in different culturing systems induced at OD_600nm_ 0.6–0.8 with 10 mM rhamnose

Culturing *E. coli* BL21 pSAR-2::sfGFP in the presence of increasing amounts of rhamnose resulted in increasing levels of fluorescence (Fig. 2a) over time. Tunability of the expression system can be observed for L-rhamnose concentrations above 0.25 mM. At the highest concentration of rhamnose (15 mM), the amount of sfGFP produced after 7 h of induction was 2.47 ± 0.12 g/L, which increased to 3.4 g/L after 24 h (Fig. 2a, c). Without induction, basal fluorescence levels corresponding to 0.23 g/L sfGFP were observed after 7 h. This suggests that the basal level of production is approximately 10% of the amount of sfGFP produced in the presence of 15 mM rhamnose. The *E. coli* BL21 pSAR-2 strain without the sfGFP insert did not show any detectable level of fluorescence at any time point. Production of sfGFP did not seem to affect cell growth significantly, as biomass yields were similar for all cultures after 7 h of growth. At this time point, for example, the *E. coli* BL21 pSAR-2 strain without sfGFP showed an OD_600nm_ value of 7.1, while sfGFP expressing cultures showed OD_600nm_ values between 6.0 and 7.0.

In addition, FACS analysis was used to determine whether there was one homogeneous cell population where all cells contributed equally to sfGFP expression. The data show that after 4 h of induction, all cells produced sfGFP proportionally to the rhamnose concentrations used, thus forming one normally distributed population (Fig. 2b). Higher concentrations of rhamnose resulted in increased levels of sfGFP fluorescence per cell, visible as a shift in fluorescence in the histograms. Cells which contain pSAR-2 but without sfGFP (negative control) did not show fluorescence and non-induced cultures showed a baseline level of fluorescence that corresponded to less than 5% of the mean fluorescence detected for cells of the 15 mM induced cultures. No major morphological differences were observed between the cultures regarding the cell size (forward scatter (FSC-A)) and granularity (side scatter (SSC-A)), indicating that growth and cell division were unaffected, and that the formation of inclusion bodies is unlikely. Taken together, this shows that the pSAR-2 vector allows for the controlled, uniform, and inducible expression of proteins upon induction with different rhamnose concentrations.

### Scalability of sfGFP expression

To test the robustness of this expression vector, *E. coli* BL21 pSAR-2::sfGFP cells were tested in different cultivation systems. Thus, cells were grown in batch cultures and induced with 10 mM of rhamnose in TPP TubeSpin® Bioreactors, shaker flasks, UltraYield™ flasks and in bioreactors. Both the TPP TubeSpin® Bioreactors and the shaker flasks delivered similar concentrations of sfGFP after 24 h of induction time, with 3.5 and 3.4 g/L sfGFP production respectively (Fig. 2c), as well as comparable yields of biomass with an OD_600nm_ of 15 and 12 for the TubeSpin® Bioreactors and the shaker flasks, respectively. In turn, the UltraYield™ shaker flasks and the Bioreactors resulted in rapid growth and high yields of sfGFP expression, resulting in optimal production already at 5.5–6.0 h after induction. With these systems, 4.9 g/L sfGFP and 5.6 g/L sfGFP were obtained in the bioreactor and in the UltraYield™ flasks, respectively (Fig. 2c). Final biomass yields were very high in both systems, with OD_600nm_ values of 21–22 for both systems. It is also important to note that bioreactor cultures were done in the absence of selection pressure (no zeocin was added during cultivation), so as to obtain insight into the stability of the vector system in the absence of antibiotics.

### Expression of antibody fragment PGT135 scFV

**Fig. 3:**
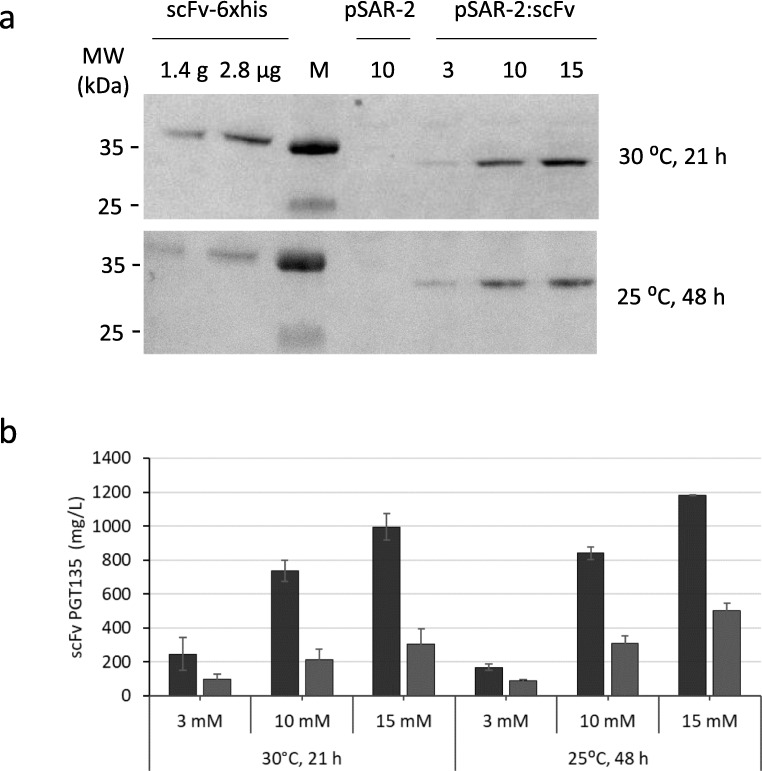
Expression of antibody fragment PGT135 scFv. PGT135 scFv is expressed by *E. coli* BL21 from the pSAR-2:scFv vector with different concentrations of rhamnose for induction, and at two different temperatures (30 °C and 25 °C) and harvest times (21 h and 48 h). **a** Representative western blot with Protein L–HRP conjugate binding to PGT135 scFv antibody fragments in the soluble protein fraction. Numbers 3, 10, and 15 indicate the amount of rhamnose added for induction in mM. *E. coli* BL21 pSAR-2 empty induced with 10 mM of rhamnose is included as a negative control. Two different known concentrations of the commercially available His_6_-tagged antibody PGT135 scFv-6xHis are included for quantification. **b** Graph showing semi-quantitative data for PGT135 scFv production in shaker flasks as determined by western blot based on two biological replicates. Black bars show PGT135 scFv in total cell fraction and gray bars in soluble protein fraction. Error bars indicate standard deviation of two biological duplicates

The next step was to assess whether the pSAR-2 vector is also suitable for expression of proteins that are typically more complex to produce, as for instance those that contains disulfide bonds and are more complex to fold. For this, a sequence coding for the HIV-1 neutralizing PGT135 scFv antibody fragment was designed and used. To allow for correct formation of functionally essential disulfide bonds, a PelB leader sequence was added N-terminally to direct the antibody fragment to the periplasmic compartment of the cell. The DNA sequence of the gene for PGT135 scFv (Genbank Accession MN146015) was codon optimized and synthesized by Invitrogen GeneArt Gene Synthesis (Regensburg, Germany) and cloned using *Nde*I/*Bam*HI into the multiple cloning sites of the vector. After transformation to *E. coli* BL21 cells, the PGT135 scFv antibody fragment was expressed in 500 mL shaker flasks, containing 100 ml of TB medium. An initial screening with a wide range of rhamnose concentrations (0.1–15 mM), different post induction cultivation temperatures (20–37 °C), and various harvest times (21 h–72 h) was performed to select the best parameters for PGT135 scFv expression. In Fig. 3, results are presented for shaker flask cultures induced with rhamnose to a final concentration of 3 mM, 10 mM, or 15 mM, where the temperature after induction was reduced from 37 to 30 °C or 25 °C, and where cells were harvested 21 h and 48 h after protein induction, respectively. Western blot analysis was performed on the total and soluble protein fractions making use of a Protein L–horseradish peroxidase (HRP) conjugate for visualization and semi-quantification using a standard (commercial scFv PGT135-His_6_-tag antibody) at a known concentration.

As expected, no protein was visible after western analysis of the *E. coli* BL21 cells with the empty pSAR-2 vector (Fig. 3a). All induced cultures containing cells with the pSAR-2::scFv vector showed expression of a protein with the expected size of 29 kDa, which was recognized by the Protein L–HRP conjugate (Fig. 3a). The commercial PGT135 scFv antibody fragment used as control had a slightly lower mobility in the SDS-PAGE gel, which was expected due to the presence of a linker sequence and His_6_-tag in this commercial antibody. In addition, the bands identified on the SDS-PAGE that have the expected size of scFv PGT135 were further corroborated by LC-MS/MS analysis. This clearly demonstrated the presence of the scFv PGT135 antibody fragment (Figure S1). No peptides were found covering the region of the PelB leader sequence, which is most likely removed upon translocation. Altogether, this confirms the identity of the visible band as our PGT135 scFv antibody fragment.

Increasing the rhamnose concentrations resulted in higher production of scFv GT135 at both tested temperatures (Fig. 3b), as was also observed during the rhamnose titration expression experiments with sfGFP. In general, expression was higher in the cultures grown at 25 °C as compared with those grown at 30 °C. Thus, the highest amount of PGT135 scFv in both soluble fraction and total cells was produced after 48 h at 25 °C in the presence of 15 mM rhamnose. By comparing in the western blot the intensity of these bands to that of a standard, the soluble fraction contained an amount of antibody equivalent to 502 ± 45 mg/L of the commercial antibody, while the total fraction contained an equivalent of 1182 ± 2 mg/L. In comparison, the highest amounts of PGT135 scFv produced after 21 h of expression at 30 °C with 15 mM rhamnose were 304 ± 88 mg/L and 996 ± 77 mg/L in the soluble fraction and total cells, respectively. Further analysis shows that the 25 °C cultures also performed better regarding solubility of the PGT135 scFv antibody fragment, since approximately 40% of antibody fragment ends up in the soluble fraction in cultures grown at 25 °C and induced with 10 or 15 mM rhamnose, as compared with approximately 30% in the equivalent cultures grown at 30 °C. Most likely, the increased solubility at lower growth temperature is caused by the slower growth kinetics and the extended period before harvest, which allows for lower production rates and more time for protein translocation to periplasm.

Growth of *E. coli* BL21 was partially reduced by expressing the scFv PGT135 antibody fragment, as observed by the slightly lower optical densities measured at the time of harvest. For example, after 48 h of induction at 25 °C, the OD_600nm_ of the cultures with the empty plasmid control strain was 27.5, while it was 26.6, 24.7, and 22.5 for the 3 mM, 10 mM, and 15 mM induced cultures expressing scFv, respectively (see Figure S2). This trend could be explained by the increased metabolic burden imposed by the recombinant protein synthesis and possibly by inclusion body formation, limiting to some extent cell growth of the *E. coli* host strain.

In conclusion, significant amounts of scFv PGT135 antibody are produced in the soluble fractions (30–40% from the total) of *E. coli* BL21 from the pSAR-2:scFv vector at the tested rhamnose concentrations (10–15 mM) at 25 °C.

### Production and purification of PGT135 scFv

To obtain sufficient purified scFv PGT135 antibody for functionality studies (HIV neutralization assays), production of PGT135 scFv was scaled up to 0.75 L bioreactors and a batch fermentation process was performed with *E. coli* BL21 cells expressing pSAR-2:scFv PGT135. Based on the results from experiments in shaker flasks, two bioreactors were induced with 15 mM L-rhamnose and run at 30 °C for 7 h and two other bioreactors were induced with 10 mM L-rhamnose and run at 25 °C for 18 h. Western blot analysis was used to semi-quantify the amount of scFv PGT135 antibody produced in the total and soluble protein fractions by comparing them with the standard commercial antibody of a known concentration. Based on this, we determined that cultures from the bioreactor performed at 30 °C contained an amount of scFv antibody equivalent to 752 ± 188 mg/L of commercial antibody in the total cell lysate, and from the bioreactor run at 25 °C the total amount of antibody in the cell lysates was equivalent to 762 ± 145 mg/L.

To extract proteins from the periplasmic fractions where the scFv PGT135 is directed, four different periplasmic extraction protocols (Ausubel et al. 1989; Dalbøge et al. 1989; Chaib et al. 1995; Rathore et al. 2003) were tested. The osmotic shock protocol (Ausubel et al. 1989) was selected as it resulted in the highest amounts of extracted soluble periplasmic scFv PGT135. Unfortunately, the extraction with this technique was still inefficient with only 4–15% of the total amount of soluble PGT135 scFv being extracted. However, these experiments corroborate that indeed scFv is secreted into the periplasmic compartment (see Figure S3B) and that it might contain the essential disulfide bonds for proper folding and therefore, functionality. To increase the recovery and to obtain enough purified scFv antibody for functionality testing, the antibody fragment was thus isolated directly from the total soluble fraction, which was obtained using commercial protein extraction *reagents* followed by centrifugation (see Materials and Methods).

The obtained soluble protein fraction of the culture from the bioreactor setup run at 30 °C contained an amount of scFv antibody equivalent to 164 ± 51 mg/L which is 22% of the total amount of antibody produced. At 25 °C, a better ratio of soluble vs insoluble scFv antibody was seen, with 30% of the total amount being found in the soluble fraction (228 ± 74 mg/L soluble scFV PGT135) (Figure S3).

**Fig. 4:**
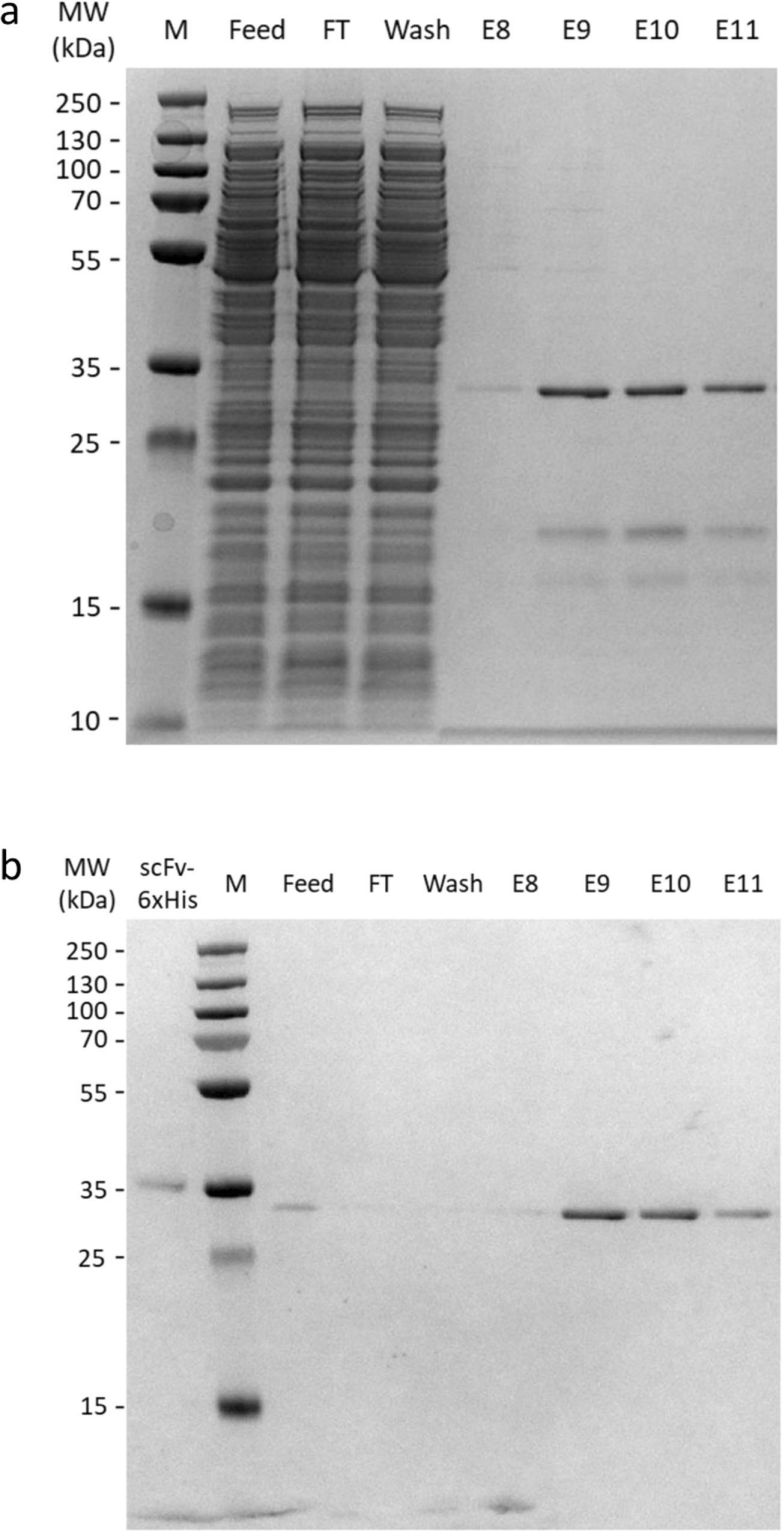
SDS-PAGE and Western blot analysis of antibody affinity chromatography purification of PGT135 scFv. Analysis of feed, flow-through (FT), wash, and elution fractions 8–11 (E8–E11) of an affinity chromatography run using Capto-L for purification of PGT135 scFv antibody fragment from the soluble protein fraction of *E. coli* BL21 cells expressing PGT135 scFv from pSAR-2 vector. **a** Representative SDS-PAGE gel showing total protein in different analyzed fractions and PGT135 scFv antibody with an expected band size of 29 kDa. Elution fractions show highly enriched PGT135 scFv after purification. **b** Representative western blot with HRP–Protein L binding to PGT135 scFv in different analyzed fractions. In the first lane, 2.8 μg of commercially available His-tagged PGT135 scFv (scFv-His_6_xHis) is included for semi-quantification. PGT135 scFv is clearly visible and semi quantified in feed and elution fractions

Affinity chromatography was performed using a Capto-L resin, which binds to the antibody’s kappa light chain and is therefore suitable for the purification of antibody fragments as scFv or Fab fragments. Initial testing with a small-scale gravity flow purification showed high affinity of Capto-L for the scFv PGT135 antibody fragment and low recovery after elution. A small-scale screening (1 ml) using different elution buffers (glycine, guanidine chloride, citrate, high salt (MgCl_2_)) at different molarities and pH conditions showed elution only at low pH (pH < 2.5) with a low molarity buffer consisting of 0.05 M glycine and 0.05 M citrate. To purify PGT135 scFv from the total soluble protein fraction from 50 mL cell culture of the bioreactor operated at 25 °C, an ÄKTA pure chromatography system was used. Elution of target protein was detected by UV absorbance at 280 nm as a sharp peak. Amounts of PGT135 scFv in feed, flow-through, wash, and elution fractions were analyzed and semi quantified by SDS-PAGE and western blot analysis as previously mentioned (Fig. 4). This shows that unbound, non-specific proteins were largely removed in the flow-through and wash fractions. The elution fractions corresponding to the 280 nm UV absorbance peak were highly enriched on PGT135 scFv (Fig. 4). However, two proteins with lower molecular weight were co-eluting with the PGT135 scFv antibody fragment, resulting in a final purity of 50–65%. Since the co-eluting proteins were only observed on the SDS-PAGE but not on the western blot (the Protein L–HRP conjugate does not bind to them), they could either be contaminating proteins or degradation products of PGT135 scFv due to the low pH used for the elution. Using this purification method, an equivalent of 27 mg of scFv antibody fragment could potentially be obtained per 0.5 L of bioreactor culture volume. At this stage, further purification optimization, for instance involving additional polishing steps to increase purity, was not required since the virus neutralization assays can be performed in relatively pure samples.

### HIV neutralization assay

To demonstrate the production of functional scFv antibody fragment, we performed assays testing the neutralizing activity of PGT135 scFv antibody fragment against a multiclade panel of HIV-1 Env pseudoviruses with known sensitivity to PGT135. For comparison, we also include a control commercially obtained His_6_-tagged PGT135 scFv and full-length PGT135 IgG antibody. The pSAR-2 expressed PGT135 scFv neutralized 9 of 19 viruses (47%) with a mean IC50 titer of 419 nM (Table 1). This activity was similar in breadth and magnitude as that observed with the commercial PGT135 scFv antibody fragment which neutralized 11 viruses (58%) with a mean IC50 titer of 147 nM. The slight differences observed between both scFv fragments can be for instance due to the purity (commercial antibody has > 90% purity). In comparison with the full-length PGT135 IgG, neutralizing activity with either scFv constructs was substantially less broad and potent when compared with PGT135 IgG which neutralized 100% of viruses with mean IC50 titer of 3.4 nM. This difference in activity may be attributed in part to the bivalent antigen binding domains of the full-length IgG molecule. Together, these data demonstrate that the pSAR-2 PGT135 scFv expressed in *E. coli* retains functional activity.

**Table 1:**
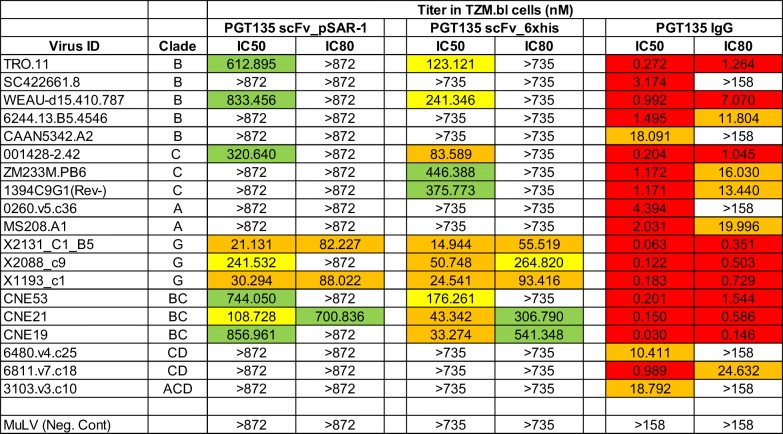
Neutralizing activity of purified PGT135 scFv antibody fragment against a multiclade panel of HIV-1 Env pseudoviruses with known sensitivity to PGT135

IC50 and IC80 titers (in nM) for neutralization potential visualized in colors (green, very low; yellow, low; orange, intermediate; and red, high)

## Discussion

Up to date, most of the recombinant mABs are produced using mammalian expression systems. However, the use of *E. coli* as an alternative host for protein production offers a more cost-effective alternative when protein glycosylation is not required, since inexpensive substrates can be used to reach high production yields in a relatively short time span. In this study, we developed an expression vector (pSAR-2) that contains a rhamnose-inducible promoter, for efficient production of high-value proteins. Utility of the vector system was shown by expressing sfGFP and HIV-neutralizing scFv antibody fragment in *E. coli.* The advantage of using the rhamnose-inducible promoter is that it is not restricted to a specific *E. coli* host, enabling the use of any standard or optimized strain for protein production. In addition, rhamnose is a cheap substrate compared with, for example, IPTG (which is used as the inducer in most *E. coli* expression systems). Protein production levels are controlled in a rhamnose concentration-dependent manner, and can be increased up to a saturation level (Giacalone et al. 2006; Wegerer et al. 2008; Hjelm et al. 2017). The pSAR-2 plasmid also allows the use of zeocin for clone selection and for maintaining selection pressure during protein production, which is advantageous as zeocin is generally accepted for use during the manufacturing of clinical products. The expression system is very stable, as illustrated by the high sfGFP production levels in a bioreactor in the absence of Zeocin, with production of up to 5 g/L in 5.5 h of cultivation; further stability studies are required to assess stability during longer production timelines. Finally, the vector was synthesized and constructed as modular build expression blocks allowing rapid exchange of diverse elements, via specific restriction sites, to adapt to the protein of interest.

Controlled expression of both sfGFP and PGT135 was achieved, with substantial yields of 2.5 g/L and 3.4 g/L sfGFP obtained after 7 h and 24 h of induction, respectively, using 15 mM L-rhamnose in shaker flasks. Expression of the HIV-neutralizing PGT135 scFv antibody fragment resulted in a high yield of 1.2 g/L total recombinant protein after 48 h of induction at 25 °C, again using 15 mM L-rhamnose in shaker flasks. Analysis of sfGFP expressing cells using a cell sorter (FACS) showed that after 4 h of induction, the level of fluorescence per cell was proportional to the concentration of L-rhamnose and that the population was homogeneous. This suggests that the P*rhaBAD* promoter offers L-rhamnose concentration-dependent control of protein production. Recent work by Hjelm and colleagues showed that the tunability of the *PrhaBAD*-mediated protein production with increasing L-rhamnose concentrations may be due more to L-rhamnose consumption rather than by regulating production rates. At lower concentrations, the inducer simply runs out earlier, leading to lower production levels. This can be resolved by using a mutant that is unable to utilize L-rhamnose (Hjelm et al. 2017). Alternatively, L-mannose could be used as inducer, as it acts as an inducer of the p*rhaBAD* promoter but is not catabolized by *E. coli* (Kelly et al. 2016).

The PGT135 scFv antibody fragment was tagged with an N-terminal PelB signal peptide for SecB-dependent secretion into the oxidizing periplasmic compartment of *E. coli* to allow for correct folding of structurally essential disulfide bridges. From the total amount of recombinant protein produced at 25 °C in shaker flasks, around 40% (0.5 g/L) was soluble. Larger scale production of scFv in non-optimized batch-process operated in 0.75 L bioreactors resulted in 0.23 g/L soluble PGT135 scFv when induced with 10 mM L-rhamnose for 18 h at 25 °C and this accounted for approximately 30% of the total amount of protein. Future optimization of the bioreactor culture conditions, for example, by performing fed-batch cultivations with different feeding strategies (e.g., using different media and supplements and controlling the time and amount of feeding) and induction regimens (e.g., time of induction, pulses of inducer), could further improve biomass yields and potentially yield even higher PGT135 scFv antibody titers. Together this result shows that the pSAR-2 plasmid is able to deliver expression of reasonable amounts of recombinant antibody both in shaker flasks as well as in small-scale fermentations.

In addition, pSAR-2 was tested in different cultivation systems (TPP TubeSpin® Bioreactors, flasks or Bioreactors). We observed that due to the small volume of the TPP TubeSpin® Bioreactors, this system is well suited for optimization of culture and expression conditions, as biomass growth and protein expression are comparable between this system and the shaker flasks. In addition, since cell growth kinetics (and biomass yields) and protein production are very similar in the UltraYield™ flasks and Bioreactors, the Ultrayield™ flasks are ideal for optimization experiments for (fed)batch cultures in the Bioreactor or as an alternative system for bioreactors when small-scale protein production is required. Altogether, these experiments demonstrate that the pSAR-2 vector is a robust scalable expression system, since it has successfully been used in different cultivation systems from the relatively small 10 mL TubeSpin® Bioreactors up to the 10 L Bioreactors with high biomass and expression yields (e.g., for sfGFP).

A major bottleneck hampering heterologous protein production in the periplasm is saturation of the Sec-translocon capacity. This would lead to a build-up of non-secreted protein in the cytoplasm, resulting in the formation of inclusion bodies consisting of insoluble protein. Incubation at 25 °C resulted in higher amounts of soluble PGT135 scFv antibody fragment in comparison with 30 °C or higher temperatures. Higher concentrations of inducer did result in higher yields of recombinant protein production, although the amount of proteins produced as inclusion bodies also increased. The best conditions for expression of scFv PGT125 involved cultivation at 25 °C for 48 h, whereby longer expression resulted in degradation and higher temperature in inclusion bodies. Leader peptides other than PelB could be explored to facilitate better protein translocation to the periplasm and thus higher soluble protein yields.

Production of tag-free PGT135 scFv antibody fragment was used as a showcase for the applicability of pSAR-2 and the p*rhaBAD* promoter for production of complex proteins. To our knowledge, this is the first time that PGT135 scFv antibody fragment is produced and purified in *E. coli* without purification tag*.* As conventional antibody purification with Protein A is only suitable for full-length antibodies, by binding to their Fc region, we made use of the binding properties of Protein L to the kappa(κ)-light chain of the antibody fragment (Rodrigo et al. 2015). Using Protein L affinity chromatography approximately 58 mg antibody per liter culture was purified with a purity of 50–60%. Although further optimization should result in higher yields and purity, this is already a reasonable amount of purified antibody fragment to expect from production in a bacterial expression system (Frenzel et al. 2013; Mesgari-Shadi and Sarrafzadeh 2017; Mesgari-Shadi et al. 2018). Initial assays showed that the PGT135 scFv fragment produced in *E. coli* was biologically active, showing HIV virus neutralization to a panel of different HIV viral strains (specially from Clade G and some from clade BC) in the same order of magnitude as the commercial His_6_-tagged PGT135 scFv antibody fragment.

The findings of this work show the applicability of the pSAR-2 expression vector as a prokaryotic platform for the controlled production of high-value and difficult-to-fold recombinant proteins. Although there are still plenty of possibilities to further improve the production yields of scFv PGT135 using the pSAR-2 expression vector in *E. coli*, the work presented already demonstrates the potential of *E. coli* as an alternative platform for the production of functional antibody fragments and new antibody fragment variants with a broad range of applications in the field of cancer, infectious diseases, immune therapeutics, or diagnosis among others.

## Electronic supplementary material

ESM 1 (PDF 712 kb)

## Data Availability

The datasets used and/or analyzed during the current study are available from the corresponding author on reasonable request.
