# Supplementary material for: A microbial expression system for high-level production of scFv HIV-neutralizing antibody fragments in Escherichia coli
Source: Appl Microbiol Biotechnol. 2019 Oct 22;103(21):8875–88. doi: 10.1007/s00253-019-10145-1 (PMC6851033; doi:10.1007/s00253-019-10145-1)
Supplement: Supplementary file 1 — (PDF 712 kb) [file 253_2019_10145_MOESM1_ESM.pdf]

Applied Microbiology and Biotechnology

SUPPLEMENTAL INFORMATION

Belonging to the manuscript:

A microbial expression system for high-level production of scFv HIV neutralizing antibody fragments in *Escherichia coli*

Marloes L.C. Petrus[1], Lukas A. Kiefer[1,3], Pranav Puri[3], Evert Heemskerk[3], Michael S. Seaman[2], Dan H. Barouch[2] , Sagrario Arias[3], Gilles P. van Wezel[1,*], Menzo Havenga [3*]

[1] Molecular Biotechnology, Institute of Biology, Leiden University, Sylviusweg 72, 2333 BE Leiden, The Netherlands

[2] Center for Virology and Vaccine Research, Beth Israel Deaconess Medical Center, 3 Blackfan Circle, Boston MA 02115, USA

[3] Batavia Biosciences B.V., Zernikedreef 16, 2333 CL Leiden, The Netherlands

*Authors for correspondence. Emails and phonenumbers: g.wezel@biology.leidenuniv.nl

+31 71 527 4310 ; m.havenga@bataviabiosciences.com +31 (0) 88 99 50 600.

Supplementary Methods

Protein sequence confirmation with MS/MS

For protein identity confirmation, an "in-gel-digestion" and LC-MS/MS method from *Shevchencko et al. 2006* was adjusted. In short, total cell protein samples from 10 mM L-rhamnose induced cultures of pSAR-2-empty and pSAR-2::scFv were separated with SDS-PAGE and stained with Coomassie Brilliant Blue according to standard procedures. SDS-PAGE bands of the expected scFv PGT135 antibody fragment size (~29 kDa) were excised from the pSAR-2::scFv sample and at similar height from the empty vector control. Excised bands were cut into small cubes (~1 mm x 1 mm), transferred to clean Eppendorf tubes and washed with milliQ. Subsequently, gel pieces were shrunk with acetonitrile and treated with 30 µl 6.5 mM DTT solution in 50 mM ammonium bicarbonate at 56 °C for 30 min. Acetonitrile was used again to shrink the gel pieces, 30 µl of 54 mM iodoacetamide was added and the sample was placed in the dark for 30 min. Three cycles of acetonitrile and 50 mM ammonium bicarbonate were used to shrink and swell the gel pieces, before proteins were digested with 30 µl of 10 ng/µl Trypsin in 50 mM ammonium bicarbonate solution for 2 h on ice and over-night at 37 °C. Afterwards, peptides were extracted from the gel pieces using 50 µl of a 1:2 mixture of 5% formic acid and acetonitrile at 37°C for 30 min. Acetonitrile was removed from the samples using a vacuum concentrator, and peptides were acidified using 70 µl of 0.5% formic acid. Acidified peptides were purified and concentrated using C_{18} stage tips [46] as described previously. The peptides were analyzed using a NanoACQUITY

UPLC system equipped with C18 Zenfit trap and analytical columns hyphenated to a SYNAPT G2-Si high-definition mass spectrometer (all from Waters) using a recently published method [47].

Isolation of periplasmic protein and small scale purifications with Protein L

Isolation of periplasmic protein is adapted from the osmotic shock protocol of Ausubel *et al*. 1989 [39]. Cells from 50 ml cultures were harvested by centrifugation (5.000 x g, 10 min, 4 °C), washed with PBS, resuspended in 5 ml of 30 mM Tris-HCl, 20% sucrose, pH 8 containing Complete Mini EDTA-free Protease Inhibitor Cocktail (Roche, Basel, Switzerland) and 10 µL of 0.5 M EDTA was added. Cells were incubated 10 min at RT with stirring and collected by centrifugation (10.000 x g, 10 min, 4°C). The supernatant is collected as periplasmic fraction 1, while the cell pellet is resuspended in 10 ml of ice-cold 5 mM $MgSO_4$ and stirred at 4 °C for 10 min to release periplasmic proteins into the buffer. The shocked cells are centrifuged (10.000 x g, 10 min, 4 °C) and the supernatant is collected as periplasmic fraction 2. Both periplasmic fractions are combined, filter-sterilized and 5 times concentrated with Amicon Ultra-15 spin filters (MW cut-off 3.000, Merck, Darmstadt, Germany).

For small scale purifications, the PGT135 scFv antibody fragment was isolated from either combined concentrated periplasmic protein fraction 1 & 2 using the affinity chromatography gravity columns with Capto L. The resin was equilibrated five times with 1 column volume of binding/wash buffer (100 mM sodium phosphate, 150 mM sodium chloride, pH 7.2) before applying 1 column volume of periplasmic protein fraction. The mixture was incubated at 4 °C for 1 h on a rocking table. The resin was loaded onto a gravity column and washed with five column volumes of binding/wash buffer. The antibody fragment was eluted with 3 column volumes of

elution buffer (0.05 M Glycine + 0.05 M citrate (pH2.0)) and neutralized immediately with neutralization buffer (1 M Tris, pH 8.0). SDS-PAGE and immunoblotting were used to determine protein concentration and purity in the eluted fractions. Elution fractions containing scFv PGT135 antibody were combined, concentrated and buffer exchanged with 1x PBS using Amicon Ultra-15 spin filters (MW cut-off 3.000, Merck, Darmstadt, Germany). Samples containing scFv PGT135 antibody were stored at -20 °C for further analysis.

Supplementary Figures

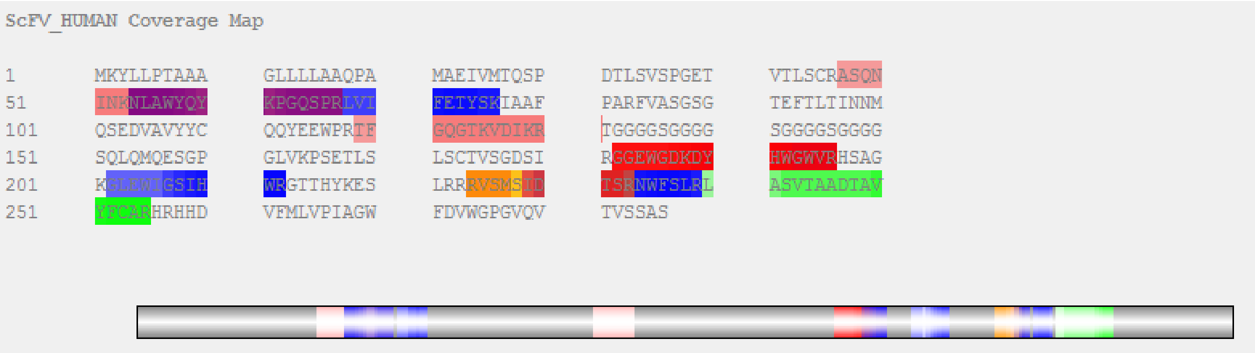

Figure S1. Protein sequence confirmation

MS protein sequencing shows that detected peptides cover 35% of the PGT135 scFv antibody fragment amino acid sequence. Regions of the protein sequence that are highlighted in color correspond to a match with a peptide (Blue), partial peptide (Red), modified peptide (Green) or partial modified peptide (Yellow). Highlights are partially transparent, so that regions where peptides overlap are visible as mixed colors on the coverage map, for example a region where a standard peptide and a partial peptide overlap would appear as purple. The original protein sequence starts with the PelB leader sequence (MKYLLPTAAAGLLLLAAQPAMA) for which no peptides were found covering this region.

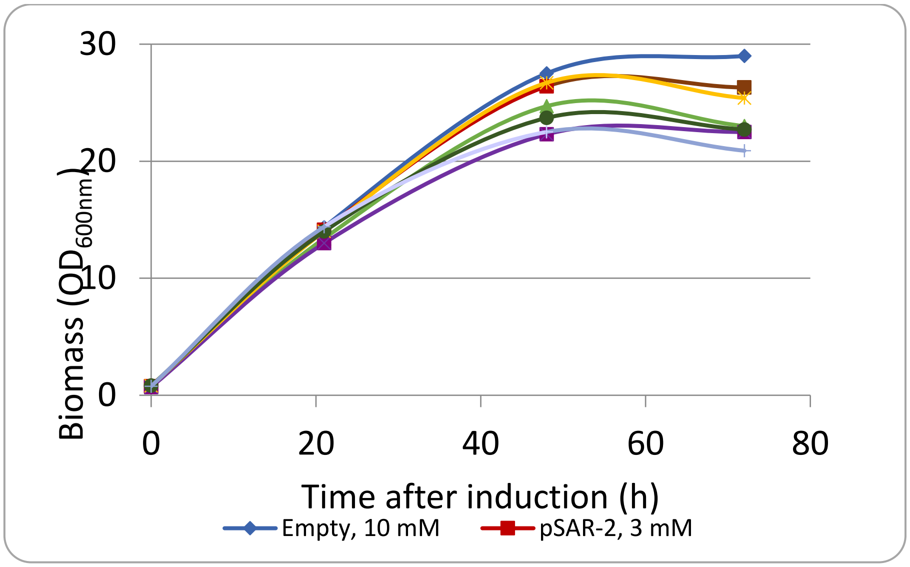

Figure S2. Growth curves of *E. coli* BL21 in shaker flasks cultures expressing scFv PGT135 from pSAR-2

Growth (OD_{600nm} measurements) of *E. coli* expressing pSAR-2:scFv PGT135 was monitored for 72 hours upon addition of L-rhamnose at different concentrations of 3 mM (A1, A2 cultures) , 10 mM (B1, B2 cultures) and 15 mM (C1, C2 cultures) and cultivation at 25 °C. Duplicates are shown. Also the empty vector was included as a negative control and induced with 10 mM rhamnose (X)

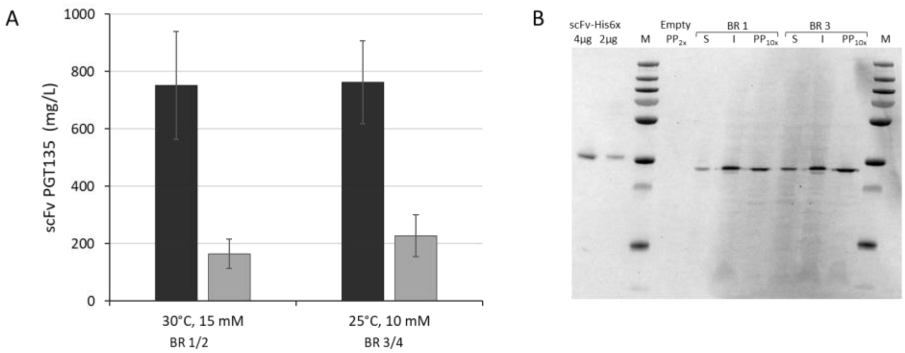

Figure S3. Scale-up and production of antibody fragment PGT135 scFv in bioreactors.

PGT135 scFv was produced in 0.75 L bioreactors in batch mode expressed from pSAR-2:scFv by *E. coli* BL21 cells. Cells were harvested at 7 h or 18 h after induction with 15 mM or 10 mM rhamnose and growth at 30 °C or 25 °C. Yields of scFv are measured in the total cell fraction (dark grey) and the soluble protein fraction (light grey) using Western blot and a standard with a known concentration. Error bars represent standard deviation between two biological replicates. B) Western Blot visualizing the scFv antibody extracted from the different fractions: (Empty PP_{2x}), periplasmic fraction from empty vector cultures 2x times concentrated, (S) soluble fraction, (I) insoluble fraction (inclusion bodies) and (PP_{10x}) periplasmic fraction 10x times concentrated.
